# Efficacy and Safety of Pharmacological Treatment in Patients with Complex Regional Pain Syndrome: A Systematic Review and Meta-Analysis

**DOI:** 10.3390/ph17060811

**Published:** 2024-06-20

**Authors:** He Zhu, Bei Wen, Jijun Xu, Yuelun Zhang, Li Xu, Yuguang Huang

**Affiliations:** 1 Department of Anesthesiology, Peking Union Medical College Hospital, Chinese Academy of Medical Sciences & Peking Union Medical College, Beijing 100730, China; 2 Department of Pain Management, Anesthesiology Institute, Cleveland, OH 44195, USA; 3 Department of Inflammation and Immunity, Lerner Research Institute, Cleveland, OH 44195, USA; 4 Cleveland Clinic, Case Western Reserve University, Cleveland, OH 44195, USA; 5 Medical Research Center, Peking Union Medical College Hospital, Chinese Academy of Medical Sciences & Peking Union Medical College, Beijing 100730, China

**Keywords:** pharmacological treatment, complex regional pain syndrome, pain relief, meta-analysis

## Abstract

Complex regional pain syndrome (CRPS) is a disabling condition that usually affects the extremities after trauma or surgery. At present, there is no FDA-approved pharmacological treatment for patients with CRPS. We performed this systematic review and meta-analysis to evaluate the efficacy and safety of pharmacological therapies and determine the best strategy for CRPS. We searched the databases, including PubMed, Embase, Cochrane, Web of Science, Scopus, and ClinicalTrials.gov, for published eligible randomized controlled trials (RCTs) comparing pharmacological treatment with placebo in CRPS patients. Target patients were diagnosed with CRPS according to Budapest Criteria in 2012 or the 1994 consensus-based IASP CRPS criteria. Finally, 23 RCTs comprising 1029 patients were included. We used the Grading of Recommendations, Assessment, Development, and Evaluation (GRADE) approach to rate certainty (confidence in evidence and quality of evidence). Direct meta-analysis showed that using bisphosphonates (BPs) (mean difference [MD] −2.21, 95% CI −4.36–−0.06, *p* = 0.04, moderate certainty) or ketamine (mean difference [MD] −0.78, 95% CI −1.51–−0.05, *p* = 0.04, low certainty) could provide long-term (beyond one month) pain relief. However, there was no statistically significant difference in the efficacy of short-term pain relief. Ketamine (rank *p* = 0.55) and BPs (rank *p* = 0.61) appeared to be the best strategies for CRPS pain relief. Additionally, BPs (risk ratio [RR] = 1.86, 95% CI 1.34–2.57, *p <* 0.01, moderate certainty) and ketamine (risk ratio [RR] = 3.45, 95% CI 1.79–6.65, *p <* 0.01, moderate certainty) caused more adverse events, which were mild, and no special intervention was required. In summary, among pharmacological interventions, ketamine and bisphosphonate injection seemed to be the best treatment for CRPS without severe adverse events.

## 1. Introduction

Complex regional pain syndrome (CRPS) is a disabling pain condition, which is distinct from other pain syndromes by the presence of autonomic dysfunction and regional inflammatory changes, with a prevalence of approximately 5.4–26.2 per 100,000 person years [1,2]. Traditionally, CRPS is classified into the following two different forms: type I, which can develop after a trauma or surgery to an extremity without a defined nerve lesion, and type II, which occurs after a defined peripheral nerve injury [3]. CRPS patients often present with allodynia, hyperalgesia, skin temperature changes, and oedema, but the signs and symptoms of CRPS will vary considerably from one patient to another; its pathophysiological mechanisms are not fully understood [4]. Current knowledge implicates multiple mechanisms, including complex contributions from a maladaptive pro-inflammatory response and a disturbance in sympathetically mediated vasomotor control, together with maladaptive peripheral and central neuronal plasticity [5,6,7]. In the past 30 years, research into CRPS and consequently understanding of the pain have grown extensively, although the mechanism remains controversial.

Diagnosis of CRPS is based on clinical manifestations according to the ‘Budapest Criteria’ by the International Association for the Study of Pain (IASP). CRPS is three or four times more common in females than in males, and the peak age of onset is between 50 and 70 years [8]. At the moment, our attention to CRPS is not enough. Epidemiological surveys have shown that many patients with CRPS do not receive appropriate treatment for their pain. The discovery of effective drug targets for CRPS is vitally imperative because of the lack of early diagnosis and understanding mechanisms. Currently, the potential pathogenic mechanisms, i.e., cytokine imbalance, neuroinflammation, and central and peripheral sensitization, underlie CRPS. In fact, a multimodal approach is recognized as the most appropriate for CRPS, and it should be started as soon as possible after diagnosis to avoid developing acute pain into chronic pain. However, there is a lack of high-quality RCTs to compare the efficacy of multimodal strategies for CRPS. At the same time, several pharmacological treatments have been proposed for CRPS, but there is a paucity of systematic reviews and meta-analyses on pharmacological therapies for CRPS [9,10]. 

Thus, we performed this systematic review and meta-analysis of RCTs to elucidate the efficacy and safety of different pharmacological approaches with CRPS.

## 2. Results

### 2.1. Eligible Studies and the Characteristics

**Figure 1:**
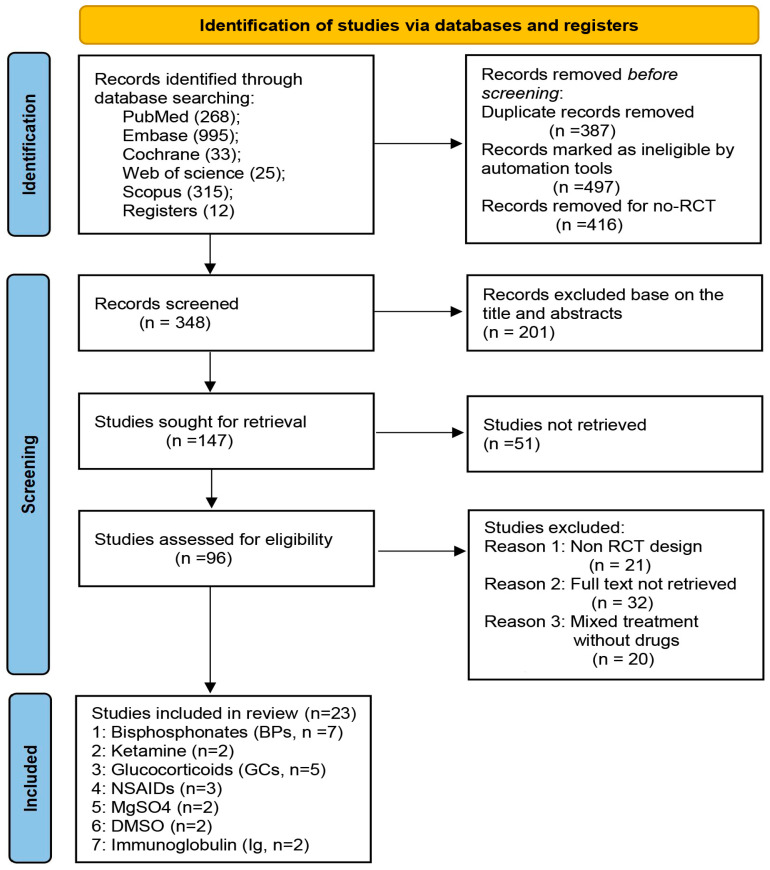
Flow chart of the study selection process. The systematic search identified 1648 articles, a total of 1300 records removed before screening, then 201 records excluded based on the title and abstracts, 51 studies were not retrieved, and 73 studies were excluded after careful assessment. Finally, 23 RCTs with 1029 patients were eligible. Abbreviations: BPs = bisphosphonates; GCs = glucocorticoids; NSAIDs = nonsteroidal antiinflammatory drugs; Ig = immunoglobulin.

The systematic search identified 1648 articles, of which 23 RCTs with 1029 patients (i.e., 641 females and 388 males) were eligible (Figure 1). All included studies assessed the analgesic effects of pharmacological treatment on CRPS patients via visual analog scale (VAS); among them, BPs (alendronate = 2; clodronate = 1; pamidronate = 2; neridronate = 2) [11,12,13,14,15,16,17], ketamine (n = 2) [18,19], GCs (prednisolone = 2; methylprednisolone = 3) [20,21,22,23,24], NSAIDs (parecoxib = 2; piroxicam = 1) [25,26,27], MgSO4 (n = 2) [28,29], DMSO (n = 2) [30,31], and immunoglobulin (Ig, n = 2) [32,33] were analyzed. As one of the important types of chronic pain, there were 377 patients (9 RCTs included) with CRPS lasting more than one year. The characteristics of the included RCTs are summarized in Table S1, Supplementary Materials.

### 2.2. Risk of Bias

**Figure 2:**
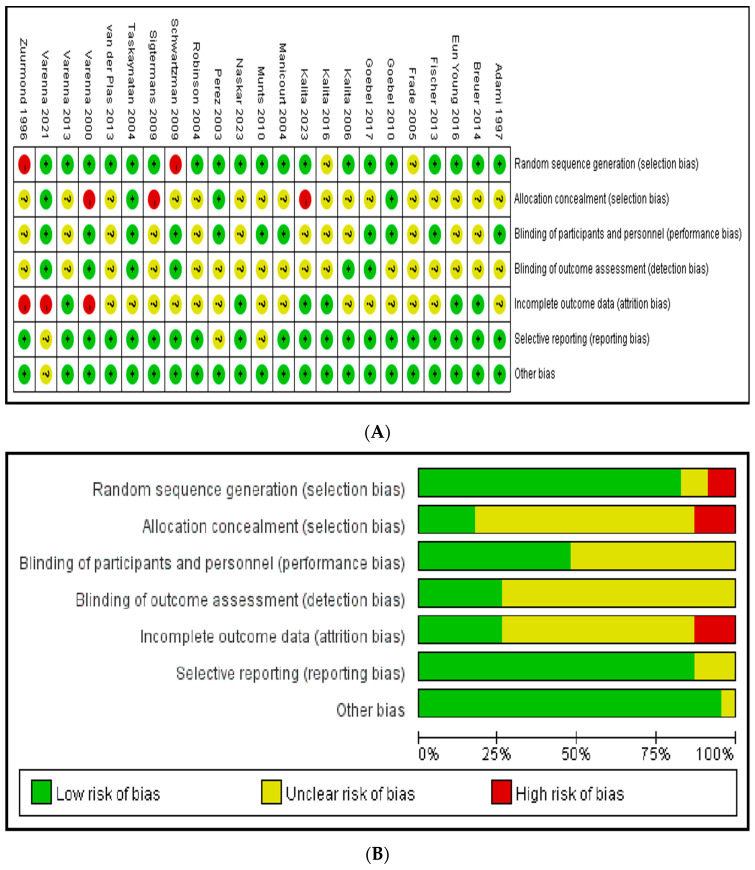
Risk of bias. (**A**) A summary table of review authors’ judgements for each risk of bias item for each study. (**B**) A plot of the distribution of review authors’ judgements across studies for each risk of bias item [11,12,13,14,15,16,17,18,19,20,21,22,23,24,25,26,27,28,29,30,31,32,33].

The high risk of bias owing to “random sequence generation”, “allocation concealment”, and “incomplete outcome data”. The risk of bias for the other domains (blinding of participants and personnel, blinding of outcome assessment, selective reporting, and other bias) was low (Figure 2). 

However, currently, there are few high-quality studies on CRPS drug therapy. It is worth noting that there may be publication bias in the included drug therapies, and the test for overall effect showed Z = 0.48 (*p* = 0.63) (Figure S1, Supplementary Materials).

### 2.3. Pairwise Meta-Analysis of Treatments on CRPS

**Figure 3:**
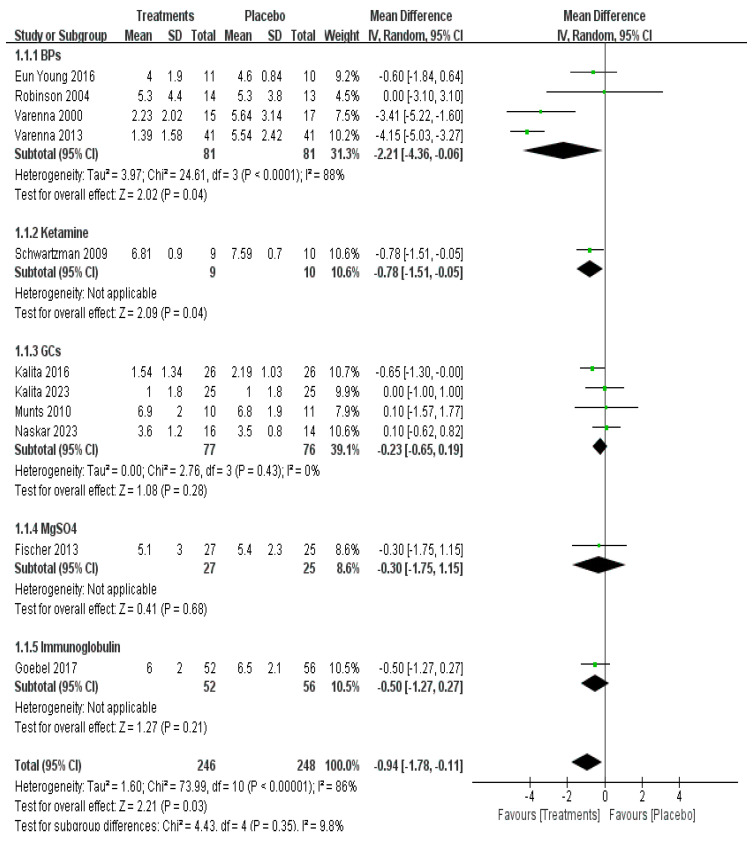
Efficacy of pharmacological treatment on long-term pain relief [12,14,15,16,19,20,21,22,23,28,33].

Direct meta-analysis of 11 studies with 494 patients showed that use of IV BPs (mean difference [MD] −2.21, 95% CI −4.36–−0.06, *p* = 0.04, moderate certainty) and IV ketamine (mean difference [MD] −0.78, 95% CI −1.51–−0.05, *p* = 0.04, low certainty) provided a statistical difference for long-term pain relief (Figure 3).

**Figure 4:**
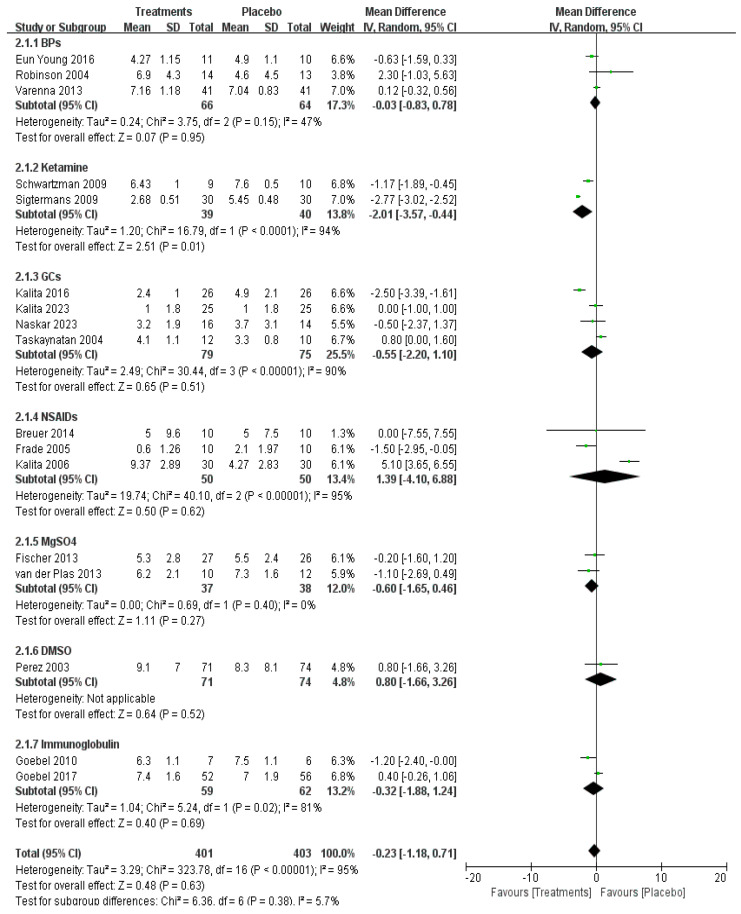
Efficacy of pharmacological treatment on short-term pain relief [14,15,16,18,19,20,22,23,24,25,26,27,28,29,31,32,33].

However, short-term pain relief within one month was investigated in 17 studies with a total of 804 patients, showing no significant difference in the overall effect (*p* = 0.63) (Figure 4). 

**Figure 5:**
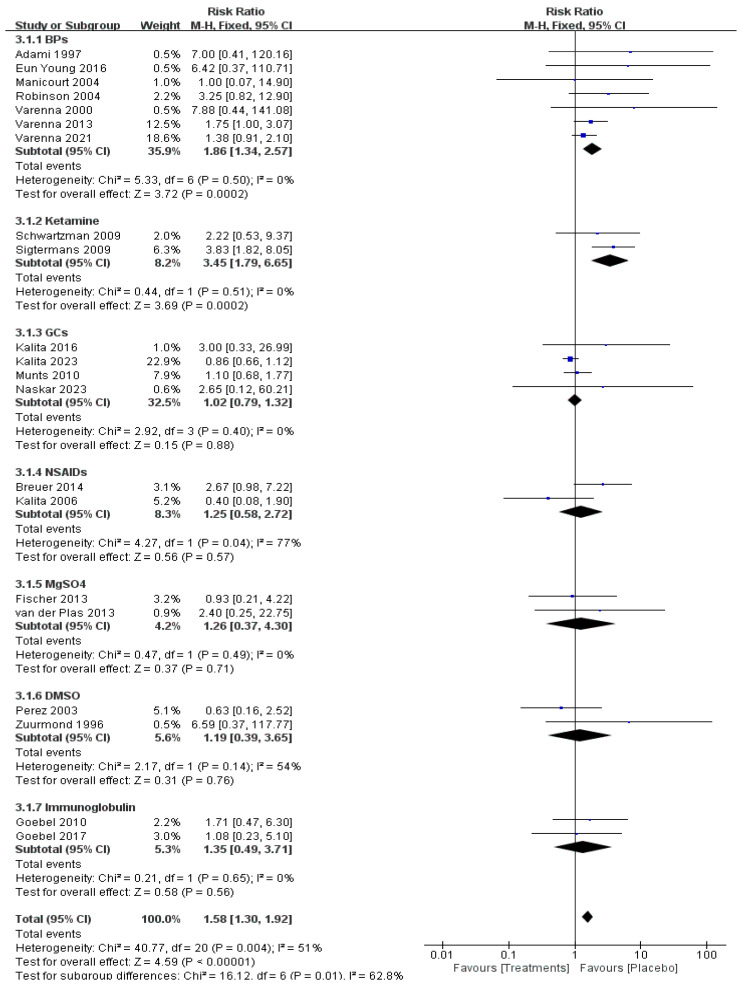
Adverse event of pharmacological treatment on CRPS [11,12,13,14,15,16,17,18,19,20,21,22,23,26,27,28,29,30,31,32,33].

Data on adverse events of relevant drugs were available in 21 RCTs with 987 patients. The BPs (risk ratio [RR] = 1.86, 95% CI 1.34–2.57, *p* < 0.01, moderate certainty) and ketamine (risk ratio [RR] = 3.45, 95% CI 1.79–6.65, *p* < 0.01, moderate certainty) showed more adverse events but no serious side effects compared with placebo (Figure 5). Common adverse effects of BPs include mild fever, malaise, and arthralgia, which are all self-limiting. Adverse effects of ketamine included dysphoria, nightmares, hallucinations, insomnia, agitation, and blurred vision.

### 2.4. Network Meta-Analysis of Treatments on CRPS

**Figure 6:**
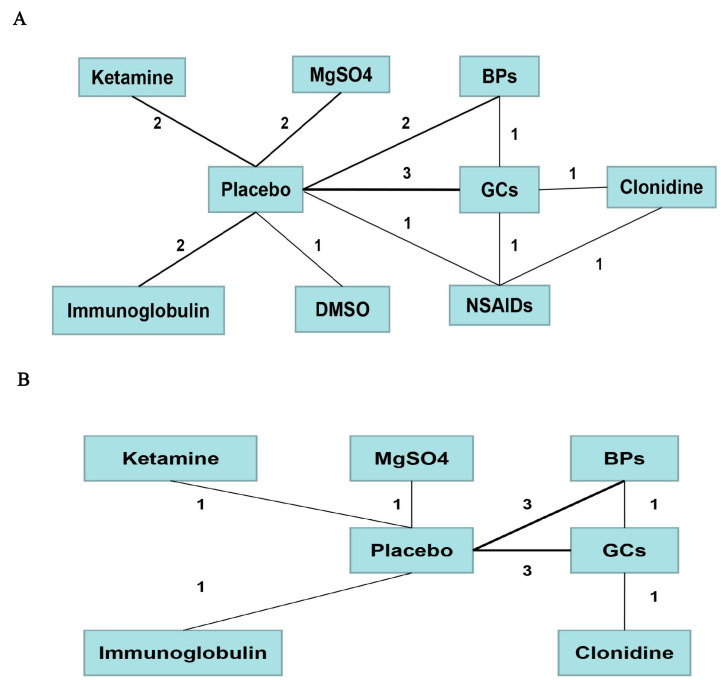
Comparison network of the included RCTs. Each line connected two pharmacological treatments from the original studies. The number on the line refers to the quality of studies comparing each pair of strategies, which were also represented by the width of the lines for the following. Part (**A**) included strategies for short-term pain relief. Part (**B**) included strategies for long-term pain relief.

We conducted a network comparison containing the above-mentioned different pharmacological strategies by establishing connections between each strategy (Figure 6). 

**Figure 7:**
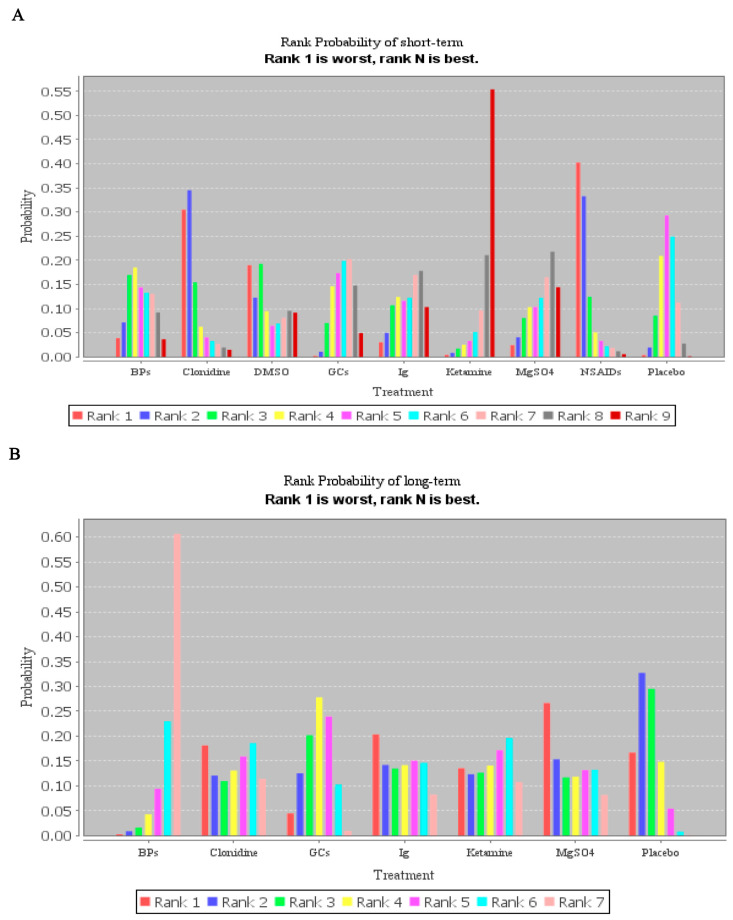
Rank probability plots of the network meta-analysis for the association of different pharmacological treatments with their outcomes. (**A**) Rank probability plots of treatment in short-term pain relief. (**B**) Rank probability plots of treatment in long-term pain relief.

Then, based on the network meta-analysis and subgroup analysis of drug treatment, the results showed the ketamine (rank *p* = 0.55) and BPs (rank *p* = 0.61) may be the best strategy for short- and long-term pain of CRPS when compared with placebo, respectively (Figure 7). However, it should be noted that in NMA estimates of all drugs against each other, there was no significant statistical difference in short-term pain relief, and only the treatment of BPs showed a difference in long-term pain relief when compared with placebo (Table S2, Supplementary Materials). Although pharmacological therapies can be ranked statistically from the best to the worst based on rank probability plots, the effects of interventions that rank higher might not be significantly different than those of interventions that rank lower.

### 2.5. Consistency and Convergence Analysis

After constructing the node-splitting models, we found that no significant inconsistency existed in this research (Table 1). Thus, the result of the consistency model was reliable. Moreover, all PSRF values of the different parameters were limited to 1, which demonstrated that this research achieved good convergence efficiency.

### 2.6. Additional Outcomes

We found the occurrence of upper/lower limb injury was 427/314 through 19 RCTs; interestingly, data suggest that CRPS can spread outside of the originally affected limb (lower with spread to lower, upper with spread to lower, and upper with spread to upper, then lower) [31]. There were 12 RCTs included in this study. Moreover, 451 patients showed that the proportion of fractures, trauma, and surgeries resulting in CRPS were 32.4%, 33.7%, and 11.3%, respectively. Of the included three studies for post-stroke CRPS [20,27,31], they showed that BPs and NSAID therapies were safe, well tolerated, and appeared as effective as GCs.

## 3. Discussion

The pathophysiology of CRPS is poorly understood; early diagnosis and treatment are deemed crucial to avoid worse outcomes [4]. Treatments (i.e., pharmacologic management, physical rehabilitation, psychological support, and interventional pain management) for CRPS are generally driven by clinician experience, as evidence-based recommendations are lacking [34]. To the best of our knowledge, no other network meta-analysis (NMA) on pharmacological treatments for CRPS has been performed. The aim of the review was to summarize the published data concerning the drug treatment of CRPS, specifically the pain relief based on VAS.

The anti-osteoclastic agents BPs have been reported to provide analgesia in a number of bone-related pains, including Paget’s disease and acute vertebral fracture. BPs have emerged as an effective treatment for CRPS pain [35,36]. It was postulated that BPs improve pain in selected sub-types of CRPS I patients through interacting with the immune system and regulating expression of nerve growth factor [33]. Our meta-analysis showed BPs provided late (beyond one month) but not early (within one month) pain relief. According to the recent Cochrane review [37], there was low-quality evidence that BPs may be effective for treating pain in CRPS-I, and it was possible that pain relief might be specific to clinical signs of osteopenia or osteoporosis.

Recent studies have demonstrated that ketamine, a strong NMDA receptor antagonist, has a growing body of clinical evidence to support the treatment of neuropathic pain, especially CRPS [38]. Our findings suggested that IV ketamine infusion can provide clinically effective pain relief in the short term. Schwartzman [19] reported that the pain relief remained statistically significant at three to four-week follow-up but did not reach significance beyond that time. Similarly, according to the study of Goebel [39], low-dose ketamine within a 4.5-day infusion sometimes produced impressive analgesia with acceptable side effects. A cross-over trial also investigated a topical 10% ketamine cream compared to a placebo cream in a mixed population of CRPS-I and CRPS-II patients, but the study did not include pain intensity as a clinical outcome; rather, they assessed the efficacy on allodynia [40].

Of note, GCs have powerful anti-inflammatory effects throughout the whole body, with COX-2 repression as one of the mechanisms of action, and high doses of GCs have been found beneficial in CRPS, especially in its early stages. According to the study of Kalita [23], prednisolone treatment has shown improvement in the Barthel Index score and modified Rankin Score along with improvement. A recent study found the efficacy of prednisolone in diabetic patients with CRPS-1, but 20 mg is not inferior to 40 mg [20]. Based on the recent review, there is evidence to support GCs in CRPS, but the ideal administration route and dose remain unclear [41]. However, in the five RCTs we included, no statistical difference was found both in short- and long-term pain relief. 

NSAIDs have been used to target the pain and inflammation underlying CRPS in both adults and pediatric patients [42,43], but the study did not find a significant difference when compared with placebo. Significant reductions of pain and sensory disturbances in acute-stage CRPS patients were found on intravenously administered magnesium in a pilot study [44]. Interestingly, in contrast to previous negative reported results from human trials of magnesium therapy for CRPS, MgSO4 for preeclampsia was associated with complete alleviation of chronic lower extremity pain [45].

Free radical scavengers such as DMSO and N-acetylcysteine (NAC) are widely used in the treatment of CRPS due to the excess free radical production generated by an overt inflammatory response to tissue injury [31,46]. However, current studies have demonstrated no evidence to support the use of DMSO, and no significant differences were found in our analysis. There is evidence for immune activation in the affected limb, peripheral blood, and cerebrospinal fluid, which suggests that modulating the immune system may alleviate CRPS. An open study demonstrated that low-dose Ig reduced pain intensity by 30% or more in approximately 50% of patients [47]. According to Andreas Goebel, 36 low doses of Ig (0.5 g/kg per dose) over 6 weeks had no clinically important effect on the intensity of pain. We first evaluated the efficacy of Ig in long-standing CRPS and found no statistical difference. Of course, it is well recognized that a prompt diagnosis is mandatory for a better outcome. Based on the available research, timely pharmacological interventions appear to be effective in treating CRPS.

Despite positive findings, we acknowledge a few limitations of this meta-analysis strictly inherent to the included RCTs. Firstly, CRPS is a relatively rare disease, and it is very difficult to acquire a sufficient sample size for statistical significance, and the effect size may be overestimated in previous meta-analyses of pharmacotherapy for CRPS. Secondly, there may be a population and publication bias because some studies only included males or females and the severity or long-standing of CRPS is different. A variety of pharmacologic agents have been studied for this disease, but most have been small and/or of low quality. Thirdly, we also note that fewer RCT studies included may exaggerate the therapeutic effect of ketamine on CRPS. Given the clinical heterogeneity between these studies, indirect comparisons may have a degree of intransitivity.

In addition, there are other drugs like cation-channel blockers (gabapentin, carbamazepine), augmentation of monoamines, tricyclic and heterocyclic drugs, nutraceuticals, topical interventions, and antihypertensives that also play an important role in the treatment of CRPS [48,49]. Thus, careful selection and prompt initiation of appropriate pharmacotherapy may optimize pain relief, and long-term RCTs are still required on this topic.

## 4. Materials and Methods

This meta-analysis was conducted according to the Cochrane Handbook, and the protocol for this review was prospectively registered in the International Prospective Register of Systematic Reviews (PROSPERO) database (registration number: CRD 42023444350). 

### 4.1. Search Strategy and Selection Criteria

We searched PubMed, Embase, the Cochrane Library, Web of Science, Scopus, and ClinicalTrials.gov until July 2023. The following search terms were used for the search of each database: “complex regional pain syndrome”, “CRPS” or “RSD”, “pharmacological treatment”, “bisphosphonates” (BPs), “ketamine”, “glucocorticoid” (GCs), “NSAID”, “MgSO4”, “DMSO”, “Immunoglobulin” (Ig), “Opioids”, “Calcitonin”, “Anesthetics”, and “Botulinum toxin”. This search was conducted without language restriction. The reference lists of the full-text studies were screened by us to determine trials that may be eligible. Two review authors independently scanned titles and abstracts. The final search took place on 30 July 2023.

The inclusion criteria were as follows: (1) RCTs; (2) patients with CRPS; and (3) evaluating efficacy and/or safety of pharmacological treatment. The exclusion criteria were as follows: (1) full texts were unavailable, and (2) incomplete raw data for the purpose of this research.

### 4.2. Data Extraction and Quality Assessment

The data extraction was independently accomplished by two authors. The Cochrane risk of bias assessment tool was used to assess the methodological quality of individual studies. This has the following five domains: selection bias, performance bias, detection bias, attrition bias and reporting bias. Each domain and the overall judgment classify the study as low, unclear, and high risk of bias. 

The visual analog scale (VAS) of short-term (within one month) and long-term (beyond one month) were evaluated as the primary outcome. Secondary outcomes were adverse events (i.e., nausea, headache, polyarthralgia, and fever) and causing factors of CRPS.

### 4.3. Assessment of Certainty of Evidence

We used the Grading of Recommendations, Assessment, Development, and Evaluation (GRADE) approach to assess the certainty of the evidence included [50]. The GRADE framework incorporates RoB, consistency, and publication bias. Certainty of the evidence usually varies from high to very low across the comparisons in meta-analysis. The GRADE approach was conducted by two independent authors to assess certainty of evidence. We then considered issues of incoherence and imprecision at the network estimate level. In this step, we used the certainty of the evidence for every treatment when compared with the same pharmacological therapy.

### 4.4. Statistical Analysis

For continuous data with the same units, the mean difference (MD) and 95% CI were calculated. For adverse events, we computed a probability ratio with the number of clear and defined events relative to the total, then pooled risk ratio (RR) and 95% CI were calculated. Results reported in median and interquartile ranges were calculated to means and SD using the methods of Wan et al. [51]. In pairwise meta-analysis, heterogeneity was assessed using I2 statistics (I2 > 50% denotes significant heterogeneity). Statistical significance was set at *p* < 0.05.

Network meta-analysis (NMA) incorporates both direct and indirect information through a common comparator to obtain estimates of the relative drug interventional effects on multiple intervention comparisons [52]. Additionally, convergence is assessed using the Brooks–Gelman–Rubin method, and a Potential Scale Reduction Factor (PSRF) close to one indicates approximate convergence has been reached [53]. Furthermore, a relevant rank probability plot could present the potential better therapeutic treatment. Node-splitting analysis was used to assess whether direct and indirect evidence on a specific node (the split node) are in agreement [54]. RevMan 5.3 was used for direct data synthesis, and the automated Aggregate Data Drug Information System (ADDIS v1.16.8) was used for network pooled estimation.

## 5. Conclusions

In summary, among drug interventions, the application of ketamine and bisphosphonates seemed to be the best treatment for CRPS when compared with placebo without severe adverse events. At the same time, high-quality and long-term RCTs are still required on this topic.

## Figures and Tables

**Table 1:** Node splitting analysis of inconsistency.

Treatment	Direct Effect	Indirect Effect	Overall	*p*-Value	GRADE
Short-term					
BPs, GCs	0.65 (−3.81, 5.24)	−1.51 (−5.97, 2.55)	−0.49 (−3.55, 2.30)	0.42	Low
BPs, Placebo	−0.90 (−4.24, 2.34)	1.39 (−3.57, 6.44)	−0.14 (−2.91, 2.43)	0.40	Moderate
Clonidine, GCs	−0.45 (−4.26, 3.53)	−6.04 (−10.67, −0.63)	−2.52 (−5.96, 1.24)	0.09	Low
Clonidine, NSAIDs	−1.52 (−5.25, 2.06)	3.96 (−1.61, 8.92)	0.30 (−3.21, 3.79)	0.09	Low
GCs, NSAIDs	5.08 (1.60, 8.57)	−0.75 (−5.08, 3.69)	2.79 (−0.57, 5.92)	0.05	Low
GCs, Placebo	0.56 (−2.10, 3.21)	−0.48 (−5.02, 4.49)	0.33 (−1.78, 2.52)	0.68	Moderate
NSAIDs, Placebo	0.08 (−8.32, 8.43)	−2.94 (−7.27, 1.23)	−2.47 (−5.97, 1.41)	0.52	Low
Long-term					
BPs, GCs	0.59 (−2.54, 3.78)	3.07 (−0.14, 5.49)	1.90 (−0.75, 4.27)	0.20	Low
BPs, Placebo	3.40 (0.77, 5.03)	0.94 (−3.08, 4.55)	2.55 (0.20, 4.43)	0.18	Moderate
GCs, Placebo	0.30 (−1.80, 2.00)	2.78 (−1.87, 6.08)	0.63 (−1.38, 2.55)	0.19	Moderate

GRADE—Grading of Recommendations, Assessment, Development, and Evaluation; Moderate—lowered for imprecision; Low—lowered two levels for very serious imprecisions.

## Data Availability

The datasets are available from the corresponding author upon reasonable request.

## Supplementary Materials

The following supporting information can be downloaded at: https://www.mdpi.com/article/10.3390/ph17060811/s1. Table S1. Main characteristics of the studies included in the review; Figure S1. Funnel plot of included RCTs on CRPS. (A) Included strategies for short-term pain relief (Egger’s regression *p* = 0.021). (B) Included strategies for long-term pain relief (Egger’s regression *p* = 0.806). (C) Included strategies for adverse event on CRPS (Egger’s regression *p* = 0.015); Table S2. League table presenting all network meta-analysis estimates of pain relief in short and long-term.

## References

[1] Bruehl S. (2015). Complex regional pain syndrome. BMJ.

[2] Harnik M.A., Kesselring P., Ott A., Urman R.D., Luedi M.M. (2023). Complex Regional Pain Syndrome (CRPS) and the Value of Early Detection. Curr. Pain Headache Rep..

[3] Popkirov S., Hoeritzauer I., Colvin L., Carson A.J., Stone J. (2019). Complex regional pain syndrome and functional nerological disorders—Time for reconciliation. J. Neurol. Neurosurg. Psychiatry.

[4] Harden R.N., McCabe C.S., Goebel A., Massey M., Suvar T., Grieve S., Bruehl S. (2022). Complex Regional Pain Syn drome: Practical Diagnostic and Treatment Guidelines, 5th Edition. Pain Med..

[5] Smart K.M., Ferraro M.C., Wand B.M., O’Connell N.E. (2022). Physiotherapy for pain and disability in adults with complex regional pain syndrome (CRPS) types I and II. Cochrane Database Syst. Rev..

[6] Zhu H., Wen B., Xu L., Huang Y. (2023). Identification of Potential Inflammation-Related Genes and Key Pathways Associated with Complex Regional Pain Syndrome. Biomolecules.

[7] Iolascon G., de Sire A., Moretti A., Gimigliano F. (2015). Complex regional pain syndrome (CRPS) type I: Historical perspective and critical issues. Clin. Cases Miner. Bone Metab..

[8] Ott S., Maihöfner C. (2018). Signs and Symptoms in 1043 Patients with Complex Regional Pain Syndrome. J. Pain.

[9] Shim H., Rose J., Halle S., Shekane P. (2019). Complex regional pain syndrome: A narrative review for the practising clinician. Br. J. Anaesth..

[10] Duong S., Bravo D., Todd K.J., Finlayson R.J., Tran Q. (2018). Treatment of complex regional pain syndrome: An updated systematic review and narrative synthesis. Can. J. Anaesth..

[11] Adami S., Fossaluzza V., Gatti D., Fracassi E., Braga V. (1997). Bisphosphonate therapy of reflex sympathetic dystrophy syndrome. Ann. Rheum. Dis..

[12] Varenna M., Zucchi F., Ghiringhelli D., Binelli L., Bevilacqua M., Bettica P., Sinigaglia L. (2000). Intravenous clodronate in the treatment of reflex sympathetic dystrophy syndrome. A randomized, double blind, placebo controlled study. J. Rheumatol..

[13] Manicourt D.H., Brasseur J.P., Boutsen Y., Depreseux G., Devogelaer J.P. (2004). Role of alendronate in therapy for posttraumatic complex regional pain syndrome type I of the lower extremity. Arthritis Rheum..

[14] Robinson J.N., Sandom J., Chapman P.T. (2004). Efficacy of pamidronate in complex regional pain syndrome type I. Pain Med..

[15] Varenna M., Adami S., Rossini M., Gatti D., Idolazzi L., Zucchi F., Malavolta N., Sinigaglia L. (2013). Treatment of complex regional pain syndrome type I with neridronate: A randomized, double-blind, placebo-controlled study. Rheumatology.

[16] Eun Young H., Hyeyun K., Sang Hee I. (2016). Pamidronate effect compared with a steroid on complex regional pain syndrome type I: Pilot randomised trial. Neth. J. Med..

[17] Varenna M., Braga V., Gatti D., Iolascon G., Frediani B., Zucchi F., Crotti C., Nannipieri F., Rossini M. (2021). Intramuscular neridronate for the treatment of complex regional pain syndrome type 1: A randomized, double-blind, placebo-controlled study. Ther. Adv. Musculoskelet. Dis..

[18] Sigtermans M.J., van Hilten J.J., Bauer M.C.R., Arbous S.M., Marinus J., Sarton E.Y., Dahan A. (2009). Ketamine produces effective and long-term pain relief in patients with Complex Regional Pain Syndrome Type 1. Pain.

[19] Schwartzman R.J., Alexander G.M., Grothusen J.R., Paylor T., Reichenberger E., Perreault M. (2009). Outpatient intravenous ketamine for the treatment of complex regional pain syndrome: A double-blind placebo controlled study. Pain.

[20] Kalita J., Pandey P.C., Shukla R., Misra U.K. (2023). Prednisolone 20 mg vs 40 mg in complex regional pain syndrome type I: A randomized controlled trial. J. Clin. Neurosci..

[21] Munts A.G., van der Plas A.A., Ferrari M.D., Teepe-Twiss I.M., Marinus J., van Hilten J.J. (2010). Efficacy and safety of a single intrathecal methylprednisolone bolus in chronic complex regional pain syndrome. Eur. J. Pain.

[22] Naskar S., Bhoi D., Garg H., Dehran M., Trikha A., Ansari M.T. (2023). A comparison of analgesic efficacy and safety of clonidine and methylprednisolone as additives to 0.25% ropivacaine in stellate ganglion block for the treatment of complex regional pain syndrome: A prospective randomised single blind study. Korean J. Pain.

[23] Kalita J., Misra U., Kumar A., Bhoi S.K. (2016). Long-term Prednisolone in Post-stroke Complex Regional Pain Syndrome. Pain Physician.

[24] Taskaynatan M.A., Ozgul A., Tan A.K., Dincer K., Kalyon T.A. (2004). Bier block with methylprednisolone and lidocaine in CRPS type I: A randomized, double-blinded, placebo-controlled study. Reg. Anesth. Pain Med..

[25] Frade L.P., Lauretti G.R., Lima I.C.P.R., Pereira N.L. (2005). The antinociceptive effect of local or systemic parecoxib combined with lidocaine/clonidine intravenous regional analgesia for complex regional pain syndrome type I in the arm. Anesth. Analg..

[26] Breuer A.J., Mainka T., Hansel N., Maier C., Krumova E.K. (2014). Short-term treatment with parecoxib for complex regional pain syndrome: A randomized, placebo-controlled double-blind trial. Pain Physician.

[27] Kalita J., Vajpayee A., Misra U.K. (2006). Comparison of prednisolone with piroxicam in complex regional pain syndrome following stroke: A randomized controlled trial. QJM.

[28] Fischer S.G., Collins S., Boogaard S., Loer S.A., Zuurmond W.W., Perez R.S. (2013). Intravenous magnesium for chronic complex regional pain syndrome type 1 (CRPS-1). Pain Med..

[29] van der Plas A.A., Schilder J.C., Marinus J., van Hilten J.J. (2013). An explanatory study evaluating the muscle relaxant effects of intramuscular magnesium sulphate for dystonia in complex regional pain syndrome. J. Pain.

[30] Zuurmond W.W., Langendijk P.N., Bezemer P.D., Brink H.E., de Lange J.J., van Loenen A.C. (1996). Treatment of acute reflex sympathetic dystrophy with DMSO 50% in a fatty cream. Acta Anaesthesiol. Scand..

[31] Perez M.R.S.G., Zuurmond A.W.W., Bezemer D.P., Kuik J.D., van Loenen C.A., de Lange J.J., Zuidhof J.A. (2003). The treatment of complex regional pain syndrome type I with free radical scavengers: A randomized controlled study. Pain.

[32] Goebel A., Baranowski A., Maurer K., Ghiai A., McCabe C., Ambler G. (2010). Intravenous immunoglobulin treatment of the complex regional pain syndrome: A randomized trial. Ann. Intern. Med..

[33] Goebel A., Bisla J., Carganillo R., Frank B., Gupta R., Kelly J., McCabe C., Murphy C., Padfield N., Phillips C. (2017). Low-Dose Intravenous Immunoglobulin Treatment for Long-Standing Complex Regional Pain Syndrome: A Randomized Trial. Ann. Intern. Med..

[34] Mangnus T.J.P., Bharwani K.D., Dirckx M., Huygen F.J.P.M. (2022). From a Symptom-Based to a Mechanism-Based Pharmacotherapeutic Treatment in Complex Regional Pain Syndrome. Drugs.

[35] Chevreau M., Romand X., Gaudin P., Juvin R., Baillet A. (2017). Bisphosphonates for treatment of Complex Regional Pain Syndrome type 1: A systematic literature review and meta-analysis of randomized controlled trials versus placebo. Jt. Bone Spine.

[36] Xu J., Yang J., Lin P., Rosenquist E., Cheng J. (2016). Intravenous Therapies for Complex Regional Pain Syndrome: A Systematic Review. Anesth. Analg..

[37] Ferraro M.C., Cashin A.G., Wand B.M., Smart K.M., Berryman C., Marston L., Moseley G.L., McAuley J.H., O’Connell N.E. (2023). Interventions for treating pain and disability in adults with complex regional pain syndrome- an overview of systematic reviews. Cochrane Database Syst. Rev..

[38] Zhao J., Wang Y., Wang D. (2018). The Effect of Ketamine Infusion in the Treatment of Complex Regional Pain Syndrome: A Systemic Review and Meta-analysis. Curr. Pain Headache Rep..

[39] Goebel A., Jayaseelan S., Sachane K., Gupta M., Frank B. (2015). Racemic ketamine 4.5-day infusion treatment of long-standing complex regional pain syndrome—A prospective service evaluation in five patients. Br. J. Anaesth..

[40] Finch P.M., Knudsen L., Drummond P.D. (2009). Reduction of allodynia in patients with complex regional pain syndrome: A double-blind placebo-controlled trial of topical ketamine. Pain.

[41] van den Berg C., de Bree P.N., Huygen F.J.P.M., Tiemensma J. (2022). Glucocorticoid treatment in patients with complex regional pain syndrome: A systematic review. Eur. J. Pain.

[42] Weissmann R., Uziel Y. (2016). Pediatric complex regional pain syndrome: A review. Pediatr. Rheumatol. Online J..

[43] Wertli M.M., Kessels A.G., Perez R.S., Bachmann L.M., Brunner F. (2014). Rational pain management in complex regional pain syndrome 1 (CRPS 1)—A network meta-analysis. Pain Med..

[44] Collins S., Zuurmond W.W., de Lange J.J., van Hilten B.J., Perez R.S. (2009). Intravenous magnesium for complex regional pain syndrome type 1 (CRPS 1) patients: A pilot study. Pain Med..

[45] Niconchuk J.A., Richardson M.G. (2019). Complete relief of CRPS-associated pain during magnesium infusion in a patient with postpartum preeclampsia. Reg. Anesth. Pain Med..

[46] Norton K.F., Furnish T.J. (2023). Perspectives on the pharmacological management of complex regional pain syndrome. Expert. Opin. Pharmacother..

[47] Goebel A., Netal S., Schedel R., Sprotte G. (2002). Human pooled immunoglobulin in the treatment of chronic pain syndromes. Pain Med..

[48] Taylor S.S., Noor N., Urits I., Paladini A., Sadhu M.S., Gibb C., Carlson T., Myrcik D., Varrassi G., Viswanath O. (2021). Complex Regional Pain Syndrome: A Comprehensive Review. Pain Ther..

[49] Iolascon G., Snichelotto F., Moretti A. (2024). An update on the pharmacotherapeutic options for complex regional pain syndrome. Expert Rev. Neurother..

[50] Brignardello-Petersen R., Bonner A., Alexander P.E., Siemieniuk R.A., Furukawa T.A., Rochwerg B., Hazlewood G.S., Alhazzani W., Mustafa R.A., Murad MH Puhan M.A. (2018). GRADE Working Group. Advances in the GRADE approach to rate the certainty in estimates from a network meta-analysis. J. Clin. Epidemiol..

[51] Wan X., Wang W., Liu J., Tong T. (2014). Estimating the sample mean and standard deviation from the sample size, median, range and/or interquartile range. BMC Med. Res. Methodol..

[52] Guo T., Ren L., Wang Q., Li K. (2016). A network meta-analysis of updated haemostatic strategies for hysterectomy. Int. J. Surg..

[53] Hou W., Ding M., Li X., Zhou X., Zhu Q., Varela-Ramirez A., Yi C. (2021). Comparative evaluation of cardiovascular risks among nine FDA-approved VEGFR-TKIs in patients with solid tumors: A Bayesian network analysis of randomized controlled trials. J. Cancer Res. Clin. Oncol..

[54] Dias S., Welton N.J., Caldwell D.M., Ades A.E. (2010). Checking consistency in mixed treatment comparison meta-analysis. Stat. Med..

