# Supplementary material for: Efficacy and Safety of Pharmacological Treatment in Patients with Complex Regional Pain Syndrome: A Systematic Review and Meta-Analysis
Source: Pharmaceuticals (Basel). 2024 Jun 20;17(6):811. doi: 10.3390/ph17060811 (PMC11206895; doi:10.3390/ph17060811)
Supplement: Supplementary file 1 [file pharmaceuticals-17-00811-s001.zip › pharmaceuticals-2967603-supplementary.pdf]

Table S1. Main characteristics of the studies included in the review

Author year	Intervention sample size (n)	Female/male	Age (years)	Disease duration	Efficacy (VAS) Short term	Efficacy (VAS) Long term	Adverse events (n)	Upper/lower limb(n)	Etiology (n) Fracture	Trauma	Surgery	Other
Bisphosphonates (7)												
Adami 1997	alendronate (10)	7/3	39-79	16±17 (weeks)	spontaneous pain decreased by over 75% in 5 patients and over 50% in 8 further patients	NA	3	12/8	6	1	NA	3
	placebo (10)	5/5	48-80	19±19 (weeks)		NA	0		7	2	NA	1
Varenna 2000	clodronate (15)	9/6	58.1±7.7	3.7±1.9 (months)	NA	2.23±2.02	3	2/13	3	4	1	7
	placebo (17)	10/7	53.4±9.0	4.2±2.6 (months)		5.64±3.14	0	2/15	6	4	1	6
Manicourt 2004	alendronate (20)	9/11	44.6±12.3	7±2 (months)	NA	The VAS was only 33% of that in the placebo group	1	0/20	3	13	4	0
	placebo (20)	12/8	45.2±12.5	8±3 (months)			1	0/20	4	13	3	0
Robinson 2004	pamidronate (14)	18/9 (all)	45(30-60)	21.6 (3-72) (months)	6.9(5.0-8.4)	5.3(4.5-8)	7	14/13	NA			
	placebo (13)				4.6(4-7.6)	5.3(4-7)	2					
Varenna 2013	neridronate	25/16	58.2±12.7	4.7±4.1	7.16±1.18	1.39±1.58	21	8/33	11	10	5	15

	(41)			（weeks）								
	placebo (41)	28/13	57±10.3	5±4.6 （weeks）	7.04±0.83	5.54±2.42	12	12/29	17	7	4	13
Eun Young 2016	Pamidronate (11)	6/5	63.09±10.53	54.91±33.02 (days)	4.27±1.15	4±1.9	3	11/0		Stroke for all		
	steroid (10)	5/5	67.5±6.95	47.9±18.25 (days)	4.9±1.1	4.6±0.84	0	10/0		Stroke for all		
Varenna 2021	neridronate (41)	25/16	59.3±10.2	4.8±4.9 (weeks)	50% reduction in VAS compared with placebo	NA	26	12/29	10	22	4	5
	placebo (37)	27/10	59.7±10.5	4.8±4.10 (weeks)			17	17/20	10	15	8	4
Ketamine (2)												
Sigtermans 2009	ketamine (30)	22/8	43.7±11.5	9.4±8 （years）	2.68±0.51	No statistical Difference (P = 0.07)	23	10/11		NA		
	placebo (30)	26/4	47.5±13.1	6.1±5.1 （years）	5.45±0.48		6	13/11				
Schwartzman 2009	ketamine (9)	9/0	38±7.6	5.9±4.1 （months）	6.43±1	6.81±0.9	4	2/7	3	4	0	2
	placebo (10)	9/1	45±10.5	7.3±7.1 （months）	7.6±0.5	7.59±0.7	2	3/7	2	2	3	3
Glucocorticoids (5)												
Kalita 2023	prednisolone 40mg （25）	10/15	51.08±8.79	9±6.43 (months)	1（1-2）	1（1-2）	19	NA		NA		

	prednisolone 20mg （25）	11/14	50.88±12.66	11.68±7.98 (months)	1（0-1）	1（0-1）	22					
Munts 2010	methylprednisolone （10）	10/0	45±7	5±2 (years)	NA	6.9±2	8	7/8	1	5	0	4
	placebo （11）	6/5	46±15	4±2 (years)		6.8±1.9	8	7/5	2	5	4	0
Naskar 2023	methylprednisolone (16)	9/7	45.8±12.8	3.3±1.5 (months)	3.2±1.9	3.6±1.2	1	16/0	10	0	0	6
	clonidine (14)	7/7	51.7±11.7	3.6±1.5 (months)	3.7±3.1	3.5±0.8	0	14/0	10	0	0	4
Kalita 2016	prednisolone （26）	12/14	56.6±12.5	9.52 ± 5.72 (week)	2.4±1	1.54±1.34	3	26/0		Stroke		
	placebo （26）	11/15	52.2±8.9		4.9±2.1	2.19±1.03	1	26/0				
Taskaynatan 2004	Methylprednisolone (12)	0/10	22.3±1.6	3.1±1.4（months）	4.1±1.1	No statistical difference	12	12/0	NA	6	NA	
	Placebo (10)	0/10			3.3±0.8		10	10/0		7		
NSAIDs (3)												
Frade 2005	parecoxib (10)	6/4	41±8	7-18months	0.6±1.26	NA	0	10/0		NA		
	clonidine	6/4	41±8		2.1±1.97		0	10/0				

	(10)											
Breuer 2014	parecoxib (10)	5/5	46.5(40-57)	5.5(4-36) (months)	5(0-9)	NA	8	10/0	4	1	3	2
	placebo (10)	5/5	51(22-69)	8(1-17) (months)	5(1-8)		3	10/0	5	3	0	2
Kalita 2006	Piroxicam (30)	10/20	56(40-70)	28(7-100)(days)	9.37±2.89	NA	2	NA	Stroke			
	prednisolone (30)	10/20			4.27±2.83		5					
MgSO4 (2)												
Fischer 2013	MgSO4(29)	27/2	47.2±12.2	23.0 (8.5–64.8)（months）	5.3±2.8	5.1±3.0	3	6/23	8	5	6	10
	placebo (27)	25/2	46.1±11.0	10.5 (5.0–26.8) (months)	5.5±2.4	5.4±2.3	3	10/17	7	9	5	6
van der Plas 2013	MgSO4 (10)	21/1	40 (29–52)	11.5 (6.0–16.0) (years)	6.2±2.1	NA	2	NA	4	3	10	5
	placebo (12)				7.3±1.6		1					
DMSO (2)												
Zuurmond 1996	DMSO（16）	8/8	47 (40, 61)	2 (1.5, 2.75)（months）	NA	2.1	3	14/2	9	7	0	0
	Placebo（15）	9/6	48 (41, 68)	2 (1 5, 2.5) (months)		0.9	0	12/3	8	7	0	0
Perez 2003	DMSO (71)	42/29	50.1±13.3	86 (54, 116)（days）	9.05±6.97	NA	3	51/20	NA			
	placebo (74)	54/20	48.9±15.4	102 (64.5, 164.5) (days)	8.31±8.13		5	52/22				

Immunoglobulin (2)												
**Goebel 2010**	**Ig (7)**	**10/3**	**41±10**	**19±8(months)**	**6.3±1.1**	**NA**	**4**	**8/7(two patients had spreading CRPS)**	**5 (fracture/ surgery)**	**4**	**5 (fracture /surgery)**	**3**
	**Placebo (6)**				**7.5±1.1**		**2**					
**Goebel 2017**	**Ig (52)**	**33/19**	**43.7±11.6**	**2.3±1.2(years)**	**7.4±1.6**	**6±2**	**3**	**NA**	**NA**			
	**Placebo (56)**	**42/14**	**41.0±12.5**	**2.5±1.2(years)**	**7.0±1.9**	**6.5±2.1**	**3**					

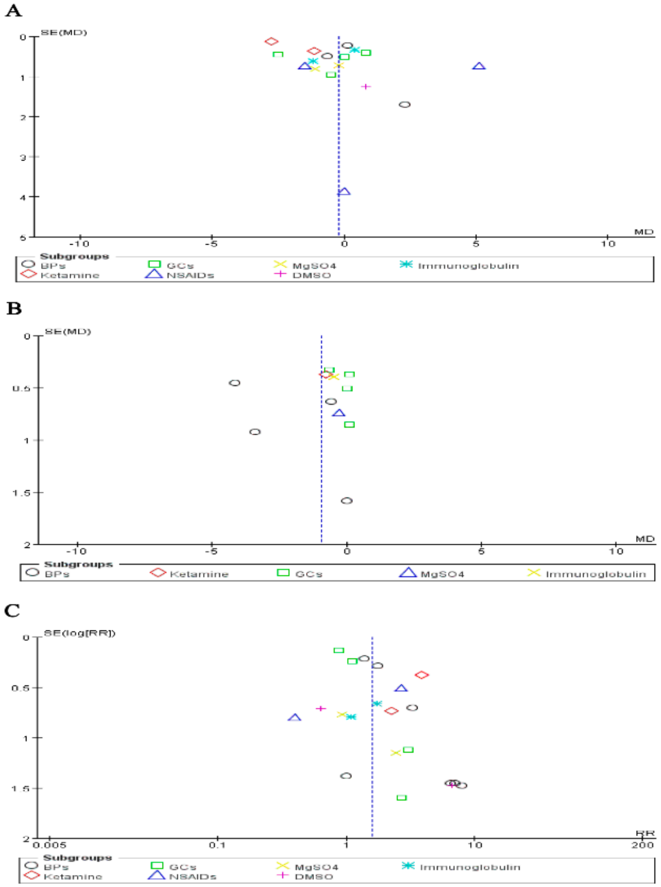

Figure S1. Funnel plot of inclued RCTs on CRPS. (A) Included strategies for short-term pain relief (Egger's regression *P*=0.021). (B) Included strategies for long-term pain relief (Egger's regression *P*=0.806). (C) Included strategies for adverse event on CRPS (Egger's regression *P*=0.015).

Table S2. League table presenting all network meta-analysis estimates of pain relief in short and long-term

Network Meta-Analysis of pain relief in short term

**BPs**								
**-2.04(-6.34,2.75)**	**Clonidine**							
**-0.61(-5.96.4.82)**	**1.39(-5.00,7.41)**	**DMSO**						
**0.49(-2.3,3.55)**	**2.52(-1.24,5.96)**	**1.10(-4.02,6.31)**	**GCs**					
**0.52(-3.39,4.68)**	**2.56(-2.56,7.38)**	**1.14(-4.38,6.75)**	**0.04(-3.61,3.72)**	**Ig**				
**2.14(-1.72,6.17)**	**4.17(-1.01,8.94)**	**2.75(-2.85,8.33)**	**1.64(-1.99,5.21)**	**1.61(-2.65,5.72)**	**Ketamine**			
**0.77(-3.16,4.93)**	**2.79(-2.41,7.73)**	**1.39(-4.18,7.11)**	**0.30(-3.43,3.94)**	**0.26(-3.98,4.50)**	**-1.36(-5.57,2.94)**	**MgSO4**		
**-2.32(-6.27,2.19)**	**-0.30(-3.79,3.21)**	**-1.68(-7.56,4.40)**	**-2.79(-5.92,0.57)**	**-2.84(-7.40,2.07)**	**-4.45(-8.95,0.40)**	**-3.09(-7.69,1.90)**	**NSAIDs**	
**0.14(-2.43,2.91)**	**2.19(-1.99,6.08)**	**0.77(-3.95,5.50)**	**-0.33(-2.52,1.78)**	**-0.37(-3.38,2.60)**	**-1.98(-4.90,0.94)**	**-0.62(-3.72,2.41)**	**2.47(-1.41,5.97)**	**Placebo**

Network Meta-Analysis of pain relief in long term

**BPs**						
**-1.85(-6.08,2.80)**	**Clonidine**					
**-1.90(-4.27,0.75)**	**-0.09(-3.85,3.54)**	**GCs**				
**-2.05(-6.07,2.35)**	**-0.24(-5.86,5.52)**	**-0.13(-4.27,4.18)**	**Ig**			
**-1.78(-5.68,2.62)**	**0.07(-5.38,5.61)**	**0.17(-3.83,4.26)**	**0.30(-4.86,5.60)**	**Ketamine**		
**-2.24(-6.32,2.34)**	**-0.42(-5.93,5.40)**	**-0.33(-4.39,3.99)**	**-0.20(-5.31,5.18)**	**-0.52(-5.56,4.76)**	**MgSO4**	
**-2.55(-4.43, -0.20)**	**-0.71(-4.84,3.62)**	**-0.63(-2.55,1.38)**	**-0.49(-4.14,3.21)**	**-0.80(-4.44,2.82)**	**-0.28(-4.20,3.41)**	**Placebo**
